# Principal Component Analysis Coupled with Artificial Neural Networks—A Combined Technique Classifying Small Molecular Structures Using a Concatenated Spectral Database

**DOI:** 10.3390/ijms12106668

**Published:** 2011-10-11

**Authors:** Steluţa Gosav, Mirela Praisler, Mihail Lucian Birsa

**Affiliations:** 1 Chemistry Department, “Alexandru Ioan Cuza” University, Carol I Bulevardul 11, Iasi 700506, Romania; E-Mails: sgosav@ugal.ro (S.G.); lbirsa@uaic.ro (M.L.B.); 2 Department of Chemistry, Physics and Environment, “Dunarea de Jos” University of Galati, Domneasca Street 47, Galati 800008, Romania

**Keywords:** GC-FTIR, GC-MS, amphetamines, PCA, ANN

## Abstract

In this paper we present several expert systems that predict the class identity of the modeled compounds, based on a preprocessed spectral database. The expert systems were built using Artificial Neural Networks (ANN) and are designed to predict if an unknown compound has the toxicological activity of amphetamines (stimulant and hallucinogen), or whether it is a nonamphetamine. In attempts to circumvent the laws controlling drugs of abuse, new chemical structures are very frequently introduced on the black market. They are obtained by slightly modifying the controlled molecular structures by adding or changing substituents at various positions on the banned molecules. As a result, no substance similar to those forming a prohibited class may be used nowadays, even if it has not been specifically listed. Therefore, reliable, fast and accessible systems capable of modeling and then identifying similarities at molecular level, are highly needed for epidemiological, clinical, and forensic purposes. In order to obtain the expert systems, we have preprocessed a concatenated spectral database, representing the GC-FTIR (gas chromatography-Fourier transform infrared spectrometry) and GC-MS (gas chromatography-mass spectrometry) spectra of 103 forensic compounds. The database was used as input for a Principal Component Analysis (PCA). The scores of the forensic compounds on the main principal components (PCs) were then used as inputs for the ANN systems. We have built eight PC-ANN systems (principal component analysis coupled with artificial neural network) with a different number of input variables: 15 PCs, 16 PCs, 17 PCs, 18 PCs, 19 PCs, 20 PCs, 21 PCs and 22 PCs. The best expert system was found to be the ANN network built with 18 PCs, which accounts for an explained variance of 77%. This expert system has the best sensitivity (a rate of classification C = 100% and a rate of true positives TP = 100%), as well as a good selectivity (a rate of true negatives TN = 92.77%). A comparative analysis of the validation results of all expert systems is presented, and the input variables with the highest discrimination power are discussed.

## 1. Introduction

The increasing popularity of Artificial Neural Networks (ANN) is explained by the large variety of research areas in which this computational method has been successfully applied [1–10]. Impressive results have been obtained with this chemometric technique for the identification and classification of molecular structures. Very frequently, ANNs are built with spectral databases, the flexibility of the method making it adequate for the analysis of nearly all types of spectra: IR [11,12], UV-VIS [13], MS [14,15], *etc*. The extensive literature research that we have performed has indicated that in most cases, ANNs have been built using a single type of spectra. This approach has the advantage that the analyst needs to record only one type of spectra, and use the dedicated ANN system, in order to automatically perform the identification of an unknown compound. However, the performances of these ANN systems cannot exceed the limits established by the amount of analytical information contained by the given type of spectra.

On the other hand, working with a hybrid database (e.g., GC-FTIR and CG-MS) usually leads to large amount of spectral data, which asks for important storage memory. Moreover, even “complementary” types of spectra, namely GC-FTIR and CG-MS contain similar information and thus include in the database important redundant information. This may affect not only the advantage of automating the process by increasing the processing time, but also the sensitivity and/or selectivity of the expert system (expressed by the false positives classification rate or by the false negative classification rate). In addition, in some fields, such as forensics, the legal consequences of the analytical result require automated detection methods with a very high correct classification rate.

Our idea had been to overcome all these problems by building an ANN spectral expert system, designed to assist the analytical toxicologists to automatically identify amphetamines or molecular structures similar to amphetamines (even if they are not present in the input data base), having as input a hybrid spectral database preprocessed by using Principal Component Analysis (PCA). The latter technique eliminates those input variables that have a lower contribution to the modeling/discrimination power of the classes of compounds, *i.e.*, stimulant amphetamines, hallucinogenic amphetamines and nonamphetamines.

**Figure 1:**
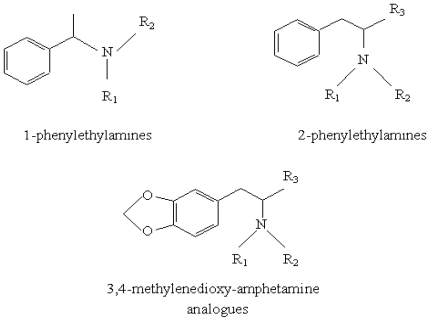
Molecular structures of the main amphetamines analogues.

As amphetamines (Figure 1) are relatively volatile substances, gas chromatography-Fourier transform infrared spectrometry and gas chromatography-mass spectrometry are the most powerful techniques applied for their identification [16]. Gas chromatography is a separation method frequently used in the case of complex mixtures such as volatile stimulants and compounds that need derivatisation (hardly volatile stimulants, narcotics, androgenic steroids, *etc*.). GC-MS with ionization source by electronic impact is used for the identification of compounds contained in the biological samples. This type of spectral analysis is also performed for doping control. GC-FTIR allows not only the identification of the eluate substances, but also their structural characterization by using the IR spectra of the compounds corresponding to the chromatographic maxima. Most of the time, GC-MS and GC-FTIR are used as complementary analytical methods for the identification of the drugs of abuse [17,18].

In this paper we present a comparative analysis of nine ANN systems: an ANN system which uses as input 100 variables selected from a concatenated spectral database representing the IR and MS spectral data and eight hybrid systems (PC-ANN networks) based on PCA and ANN which have a different number of input variables, *i.e.*, from 15 to 22 PCs. In order to obtain the scores of PCs we have applied PCA technique upon those 100 concatenated variables. This computational process has yielded, from an analytical point of view, a better reproducibility of the identification process. An optimization process has been performed in order to improve the analytical efficiency in terms of class identity assignment, by eliminating the redundant information from the spectral concatenated database and maintaining into the system the spectral information needed to maximize the modeling power. This has resulted in an enhanced data-processing speed, the selected expert system being able to identify an unknown compound in seconds. We have also determined the optimum number of PCs, by comparing the PC-ANN systems performances in classifying amphetamines according to their biological activity (stimulants or hallucinogens). The results of validation show that the 18PC_IR-MS-ANN network, that uses as input 18 PCs, identifies all the positives, has no unclassified compounds and has classified only six false positives, of which three compounds (benzylephedrine, ephedrine and methadone) are stimulants used in legitimate pharmaceutical preparations.

In addition, we present a spectroscopic analysis of the most important input variables (IR and MS spectral data) in the case of 18PC_IR-MS-ANN network, in order to identify the type of spectral information that is essential for the expert systems, in order to obtain the best analytical efficiency in the case of small molecular structures such as amphetamines.

## 2. Experimental

### 2.1. Input Database

The experimental conditions in which the GC-FTIR spectra were recorded have been presented in a previous paper [19]. The obtained reference spectra were stored in a digital library after normalization. A spectrum was normalized by dividing the intensity of the absorptions by the intensity of the strongest peak. As a result, the intensity of the latter becomes equal to 1 in the normalized spectrum. The scan range was from 4000 to 580 cm^−1^. All spectra in the library were reduced in size by eliminating the spectral windows where the compounds in the database have no IR absorptions. Hence, data ranged from 3745 to 2555 cm^−1^ and from 1995 to 605 cm^−1^. The resulting 259 wavenumber intervals of 10 cm^−1^ yielded a data matrix with 103 × 260 entries, as the database was formed using the infrared spectra of 103 forensic substances such as drugs of abuse (mainly central stimulants, hallucinogens, sympathomimetic amines, narcotics and other potent analgesics), precursors, or derivatized counterparts. The samples represent reference standards and laboratory synthesized compounds. In our case, classification experiments were carried out on 103 spectra of 15 stimulant amphetamine analogues (class code M), 5 hallucinogenic amphetamine analogues (class code T) and 83 nonamphetamines (class code N).

The mass spectra (electron impact ionization) of the 103 compounds included in the GC-MS database were imported from general MS libraries (NIST mass spectral database, AAFS spectral library, and an in-house-made MS library). The spectra from all databases were recorded in standard conditions. The MS spectra range from *m*/*z* 12 to 260.

### 2.2. Development of the Hybrid ANN Systems Using GC-FTIR and GC-MS Data

The architecture of all ANN and PC-ANN systems presented in this paper consists of three layers of neurons or nodes, which are the basic computing units: the input layer, one hidden layer and the output layer. The output layer has three nodes, one for each class of the modeled compounds namely stimulant amphetamines—class M (amphetamines with a monosustituted aromatic cycle), hallucinogenic amphetamines—class T (amphetamines with a trisubstituted aromatic cycle) and nonamphetamines—class N). The back-propagation algorithm was used for training the networks. We have adopted the sigmoid function as transfer function (activation) for all neural networks. The main network parameters (the number of nodes in the hidden layer, the learning rates, and the momentum) have been optimized by a trial-and-error process. The networks were built using the Easy NN plus software [20].

**Figure 2:**
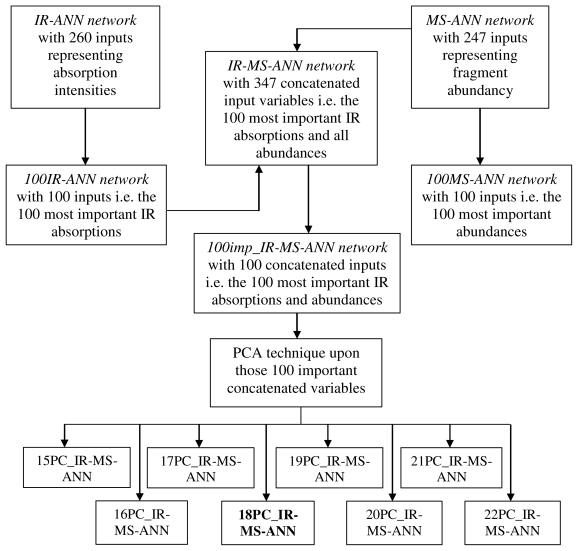
Steps performed in order to obtain the 100imp_IR-MS-ANN and then the PC-ANN networks.

We have built seven artificial neural networks aiming the automatic assignment of the class identity in the case of amphetamines. First, we have built the IR-ANN and the MS-ANN networks, that are using as inputs all the absorptions or abundancies measured in the spectra of the compounds in the database. IR-ANN system includes in its database 260 input variables representing absorption intensities measured 10 cm^−1^ apart. The MS-ANN system has 247 input variables, representing fragment abundancy. The second step has been to optimize these systems in terms of stability and analysis duration, by improving the sample/variable ratio according to the variable importance criterion. The importance analysis is a way to measure the influence of each input upon the next (hidden) layer in the network. The absolute importance of an input variable (input node) is the sum of absolute weights of the connections among this input node and the nodes of the hidden layer. As a result, we have obtained the 100IR-ANN and 100MS-ANN systems, which have an input of only 100 variables (Figure 2), *i.e.*, the 100 most important IR absorptions, and the 100 most important abundances respectively.

The third step consisted of building an IR-MS-ANN network (Figure 2) which uses a hybrid input database, formed by the 100 most important IR absorptions and all abundances (247). We chose these input variables for the IR-MS-ANN system because they have the best modeling or discrimination power. The 100IR-ANN and MS-ANN networks, which use as input variables the 100 most important IR absorptions, and the 247 abundances respectively, have the best values for the validation parameters [21].

The fourth step consisted of optimizing the IR-MS-ANN network by selecting the input variables using the importance criterion. This variable selection has been applied in order to increase the data-processing speed, to optimize the sample/variable ratio and to improve the efficiency of the class identity assignment. Using the selected input variables in function of their importance, we have built the 100imp_IR-MS-ANN network, which has 100 concatenated input variables representing the 100 most important IR absorptions and abundances (Figure 2).

The training set of the IR-ANN and 100IR-ANN networks consists of 29 samples: 7 stimulant amphetamines, 5 hallucinogenic amphetamines and 17 nonamphetamines. The remaining 130 samples were included in the validation set. The stimulants and hallucinogens included in the training set are the following: *N*-ethylamphetamine, amphetamine, methamphetamine, *N*-*n*-propylamphetamine, β-phenylethylamine, α-phenylethylamine and *N*-methyl-α-phenylethylamine, and 3,4-methylenedioxyamphetamine, 3,4-methylenedioxymethamphetamine, 3,4-methylenedioxy-*N*-ethylamphetamine, *N*-methyl-1-(3,4-methylenedioxyphenyl)-2-butanamine and 1-(3,4- methylenedioxyphenyl)-2-butanamine. The stimulant and hallucinogenic amphetamines of the training set have been selected in order of the similarity of their spectra with those of the parent compounds (amphetamine for the stimulants and 3,4-methylenedioxyampetamine for the hallucinogens). The nonamphetamines included in the training set were selected randomly from the IR database: bemegride, β-butyrolactone, cadaverine and its HFB-derivate, codeine and its pentafluoropropionic (PFP)-derivate, caffeine, γ-butyrolactone, the trimethylsilyl (TMS)-derivate of γ-hydroxy butyric acid, the TMS-derivate of γ-hydroxy valeric acid, γ-valerolactone, nicotamide, piracetam, putrescine, dextromoramide, nicotine and prolintane.

The MS-ANN and 100MS-ANN networks have the same training set (7 stimulants, 4 hallucinogens and 17 nonamphetamines) and validation set (75 samples). The stimulant and hallucinogenic amphetamines from the training set are: α-phenyletilamine, amphetamine, mephentermine, methamphetamine, *N*-ethylamphetamine, *N*-methyl-α-phenylethylamine and *N*-n-propylamphetamine, and 3,4-methylenedioxyamphetamine, 3,4-methylenedioxymethamphetamine, 3,4-methylenedioxy-*N*-ethylamphetamine and 1-(3,4-methylenedioxyphenyl)-2-butanamine. The nonamphetamines included in the training set are following: bemegride, diethylpropion, cadaverine, fencamfamine, codeine, 1-phenyl-2-propane, caffeine, γ-butyrolactone, methylphenidate, norephedrine, γ-valerolactone, nicotamide, nicotine, dextromoramide, yohimbine, morphine and lidocaine. The criterion used for the selection of the stimulant and hallucinogenic amphetamines for the training set is the same with that above-mentioned in the case of IR database.

The training set used by the IR-MS-ANN and 100imp_IR-MS-ANN networks is formed by the 7 stimulants, 4 hallucinogens and 17 nonamphetamines. The validation set contains 75 samples. The stimulant and hallucinogenic amphetamines from the training set are: α-phenyletilamine, amphetamine, β-phenyletilamine, methamphetamine, *N*-ethylamphetamine, *N*-methyl-α-phenylethylamine and *N*-n-propylamphetamine, and 3,4-methylenedioxyamphetamine, 3,4-methylenedioxymethamphetamine, 3,4-methylenedioxy-*N*-ethylamphetamine and 1-(3,4-methylenedioxyphenyl)-2-butanamine. The nonamphetamines included in the training set are the following: bemegride, diethylpropion, cadaverine, fencamfamine, codeine, 1-phenyl-2-propane, caffeine, γ-butyrolactone, methylphenidate, norephedrine, γ-valerolactone, nicotamide, nicotine, dextromoramide, yohimbine, morphine and lidocaine.

### 2.3. Building the PC-ANN Systems Using GC-FTIR and GC-MS Data

We built eight PC**-**ANNs aiming to achieve automatic assignment of the class identity of amphetamines. First, we performed the PCA analysis upon those 100 important hybrid variables (IR absorptions and MS abundances) selected by the procedure presented in paragraph 2.2. The data was preprocessed using mean-centering and autoscaling. The latter method did not provide a better explained variance, probably because the spectra in the database were recorded for standard samples and thus did not contain spectral features belonging to impurities. The scores obtained with mean-centered spectra were used as input for the PC-NN systems. Usually, the PCA technique is applied to the beginning of a data analysis in order to reduce the dimensionality of database and at the same time to retain the most relevant information from all data. Thenceforth the scores of PCs can be used as inputs for an ANN network. In our case the results presented in a previous paper [22] show that applying PCA technique on all input data before ANN network do not yield better results.

The second step consisted of building eight PC-ANN networks, which use as input a different number of variables: 15 PCs, 16 PCs, 17 PCs, 18 PCs, 19 PCs, 20 PCs, 21 PCs and 22 PCs respectively (Figure 2). The training and validation sets used by the PC-ANN networks are the same with those above-mentioned for the 100imp_IR-MS-ANN network.

**Figure 3:**
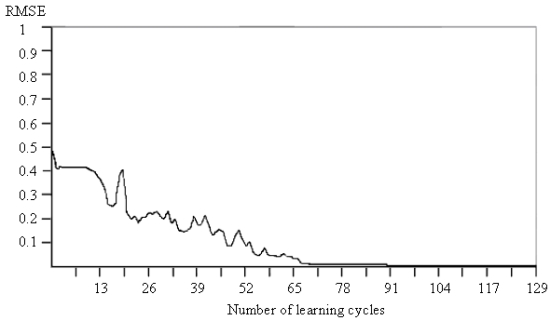
The root-mean-squared error (RMSE) of training *vs.* the number of learning cycles for the 18PC_IR-MS-ANN network.

The artificial neural networks have been programmed to stop the training process when the average error of training drops below the target error. The target error was set up at 0.01 for all ANN systems. In the case of the 18PC_IR-MS-ANN network, the convergence was touched after 129 training cycles (Figure 3). For this graph, the cycles axis is nonlinear.

## 3. Results and Discussion

### 3.1. Analysis of the Validation Results of All Artificial Neural Networks

In order to compare the efficiency of the networks, we have performed the validation process by using all the samples in the database. The validation method was full cross-validation (leave-one-out), as the number of samples in the database was relatively small. In order to evaluate the performance of the PC-ANN systems, several figures of merit for the classification were calculated: the rate of true positives (TP), of true negatives (TN), of false positives (FP), of false negatives (FN), of classification (C), and of correctly classified samples (CC). Their values are presented in Table 1. The number of unclassified compounds, of false positives and of false negatives obtained by each network, is mentioned in Table 2.

By comparing the figures of merit of the eight PC-ANN systems, we can see that all networks have a very good sensitivity (TP = 100%), with the exception of 16PC_IR-MS-ANN system, which has TP = 95%. Regarding the rate of classification, there is only one network (18PC_IR-MS-ANN) which classifies all the compounds from database (C = 100%).

Analyzing all the validation results, we arrive at the conclusion that 18PC_IR-MS-ANN is the best performing network. The 18PC_IR-MS-ANN network has a good selectivity, the rate of true negatives being TN = 92.77%. In general, the TN rate is smaller than the TP rate due to the fact that the compounds belonging to the N class have very different molecular structures, and thus very different FTIR and MS spectra. As a result, modeling the class of nonamphetamines is much more difficult than in the case of amphetamines.

The challenge which appears during the optimization process of a network is the fact that the improvement of selectivity determines, in many cases, a decrease of the others validation parameters. Thus, the idea of using a concatenated input database (FTIR and MS spectra), of the importance selection criterion and of the PCA technique for the ANN system (Figure 2) has led to an improvement of selectivity (TN = 92.77% for 18PC_IR-MS-ANN network) of the network keeping in the same time a very good sensitivity (TP = 100%) and a very good rate of classification (C = 100%).

We can see that 20PC_IR-MS-ANN has a slightly better rate of true negatives (TN = 93.67%) than 18PC_IR-MS-ANN (TN = 92.77%). However, 20PC_IR-MS-ANN is not better than 18PC_IR-MS-ANN, because 20PC_IR-MS-ANN is able to classify a smaller number of compounds (C = 96.12%) and thus the absolute number of true negatives found by 18PC_IR-MS-ANN is larger than those found by 20PC_IR-MS-ANN.

Finally, comparing the performances of 18PC_IR-MS-ANN and 100imp_IR-MS-ANN, we observe that the validation parameters have nearly the same values. Nevertheless, 18PC_IR-MS-ANN has several advantages in comparison with 100imp_IR-MS-ANN: the first network is able to classify all the compounds (C = 100%) and yields six false positives, while the latter network has a smaller classification rate (C = 98.05%, two compounds being unclassified), and yields five false positives (see Table 2). In addition, 18PC_IR-MS-ANN works faster than 100imp_IR-MS-ANN. In conclusion, 18PC_IR-MS-ANN is the best expert system of all the networks presented in this paper.

### 3.2. Discussion on the False Positives Obtained by 18PC_IR-MS-ANN

**Figure 4:**
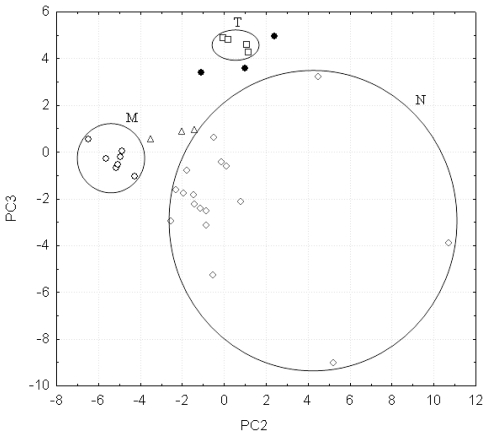
Score plot for the training set (series ○ for stimulant amphetamines, series □ for hallucinogenic amphetamines and series ⋄ for nonamphetamines), and for the false M positives (series ▵) and false T positives (series ●).

In order to find an explanation why 18PC_IR-MS-ANN classified several compounds as false positives, we have plotted the PC3 scores in function of PC2 scores for the compounds forming the training set and the false positives (Figure 4).

We have chosen PC3 and PC2 for this analysis because these PCs are the most important inputs (Table 3) from the point of view of their modeling and discrimination power. The 18PC_IR-MS-ANN network has determined six compounds as false positives, out of which three compounds are false M positives (stimulants) and other three compounds are false T positives (hallucinogens). We can see in Figure 3 that the false M positives, represented by triangles, are close to the cluster of stimulants (M). The false T positives, represented by balls, are close to the cluster of hallucinogens (T). This distribution in the n-dimensional space may be the explanation of the fact that 18PC_IR-MS-ANN provides six false positives. In addition, we should mention that the false M positives (namely benzylephedrine, ephedrine and methadone) are less dangerous stimulants, and are also found in legitimate pharmaceutical preparations. The false T positives are metoxyfenamine, safrol and tripelenamine.

It is worth emphasizing the fact that both principal components (PC3 and PC2) perform a very good separation between the compounds belonging to the M and T classes, while the separation between the M and N classes on one hand and the T and N classes on another, is attributed only to an principal component, *i.e.*, PC2 in the case of M and N classes and PC3 in the case of T and N classes (see Figure 4). As the stimulants and hallucinogens cluster in small groups (see Figure 4) we can conclude that PC2 contributes also to the discrimination between the T and N classes and PC3 distinguishes between the M and N classes. However, in this situation PC2 and PC3 have a much lower discrimination power than in the above-mentioned situation.

### 3.3. Spectroscopic Analysis of the Most Important IR Absorptions

**Figure 5:**
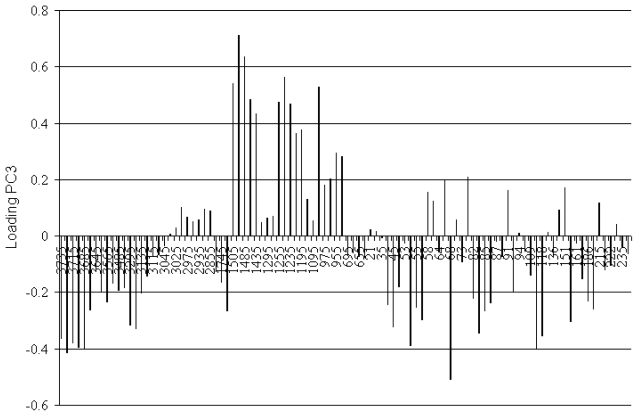
Loading plot identifying the variables (IR absorptions and *m*/*z* fragment ions) with the most important discrimination power.

The spectral domains with the highest influence on the modeling and discrimination power of 18PC_IR-MS-ANN are shown in Figure 5, which represents the loadings of the variables (IR absorptions and *m*/*z* fragment ions) for the most important PC, *i.e.*, PC3 (Table 3). The loading of a variable on a PC reflects both how much the variable contributed to that PC, and how well that PC takes into account that variable’s variation over the data points.

A first remark regarding the loadings of variables (IR absorptions and *m*/*z* fragment ions) presented in the Figure 5, is that the loadings of many IR absorptions from the spectral domain of 1505–935 cm^−1^ have higher values than those of *m*/*z* fragment ions. Therefore, we may conclude that the IR absorptions bring more important information to the expert system than the *m*/*z* fragment abundancies. However, we should not disregard the share of information given by the MS spectra and its contribution to the enhancement of the quality of the class identity assignment.

**Figure 6:**
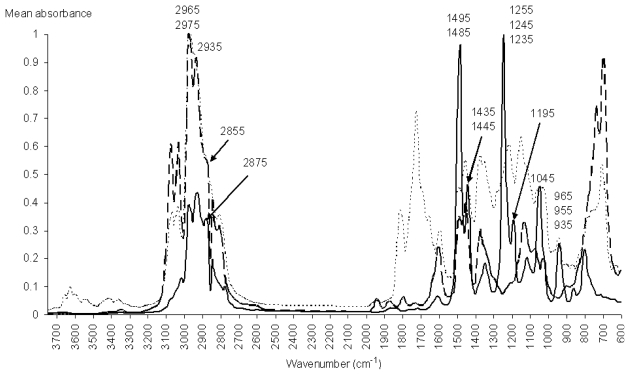
Mean spectrum of the modeled hallucinogens (___), of the modeled stimulants (_ _ _ _), and of the modeled nonamphetamines ( 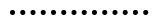), from 605 to 3745 cm^−1^.

In order to perform a spectroscopic analysis of the most important IR absorptions, we have plotted the mean spectrum of the modeled stimulants, the mean spectrum of the modeled hallucinogens, and the mean spectrum of the modeled nonamphetamines (Figure 6), in comparison with the most important IR absorptions (as shown in Figure 5).

**Figure 7:**
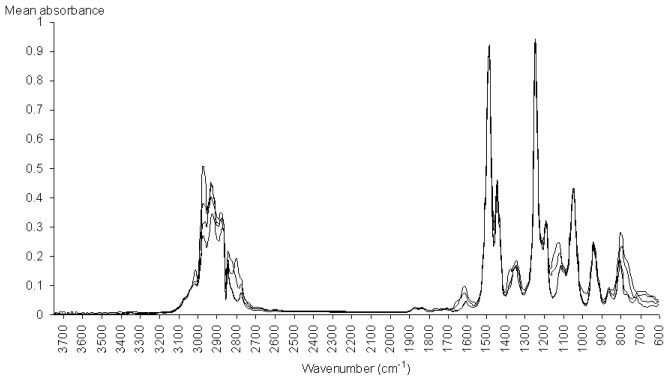
The GC/IR spectra of modeled hallucinogenic amphetamines.

The IR absorptions with the greatest contribution to PC3 are 1485 cm^−1^, 1245 cm^−1^ and 1045 cm^−1^, their loadings being larger than 0.5. All these important wavenumbers are very stable absorption peaks, from the point of view of their intensity, position and shape in the IR spectra of hallucinogens (Figure 7).

The absorption band recorded between 1520 cm^−1^ and 1400 cm^−1^ corresponds to the degenerated double stretching vibration of the aromatic C–C bond, which appears in the GC-FTIR spectra of stimulants and hallucinogens [23,24]. The absorption peaks present in this spectral domain are 1485 and 1445 cm^−1^. In the case of stimulants, these peaks have nearly the same intensity. In the case of hallucinogens, the 1485 cm^−1^ peak is much stronger than the 1445 cm^−1^ peak. In conclusion, the 1485 cm^−1^ absorption peak seems to have a major contribution to the modeling power of 18PC_IR-MS-ANN for the hallucinogenic amphetamines. On the other hand, in this spectral domain, the stimulants and the nonamphetamines have weaker absorptions than the hallucinogens, a fact which indicates that this band has an important contribution to the ability of the system to discriminate between hallucinogens and compounds having the M or the N class identity.

In the case of hallucinogens, the presence of C–O groups in the para and orto’ positions of the phenyl ring correspond to the 1245 cm^−1^ peak, which is associated with the stretching vibrations of the C–O bond [24]. Another absorption peak from the IR spectrum of hallucinogens that has a significant loading is 1045 cm^−1^, peak which is associated with the breathing vibration of the disubstituted phenyl ring [24]. The analysis has shown that another important absorption is the 935 cm^−1^ peak, which is specific to the out-of plane CH vibrations of the disubstituted phenyl ring present in the molecules of the hallucinogens. These three peaks, *i.e.*, 1245, 1045 and 935 cm^−1^ (Figure 7), are very stable, their position and intensity therefore contributing significantly to the modeling power of the system in the case of the class of hallucinogens, as well as to the discrimination between hallucinogens and stimulants.

The peaks found in the 2975–2855 cm^−1^ spectral region are associated with the C–H stretching vibrations of the aliphatic groups, and are present in the mean spectra of the stimulants, as well as in the spectra of hallucinogens. In this spectral region, there are many important wavenumbers (2975, 2965, 2935, 2875 and 2855 cm^−1^) with positive loadings, their values ranging between 0 and 0.2. As the stimulants have stronger absorptions than the hallucinogens here, we may conclude that this spectral region ensures the modeling of the class of stimulants, as well as the discrimination between the stimulants and hallucinogens.

### 3.4. Spectroscopic Analysis of the Most Important *m/z* Fragment Ions

The very good performances of the 18PC_IR-MS-ANN network may be explained by the fact that it has additional input information, represented by the GC-MS spectra of the compounds. The most important fragment ions are *m*/*z* = 58, 72, 65, 91 and 77 (Figure 5). The fragment ions *m*/*z* = 65 and 77 have the larger loadings of all fragments (about 0.2).

Analyzing the GC-MS spectra of all amphetamines [25], we found that the most frequent cleavage is the breakage of the C–C bond neighboring the nitrogen atom. This preferential cleavage rule leads to the formation of the biggest and the most stable fragment. The cyclopentadienyl (*m*/*z* = 65) and the benzyl cations (*m*/*z* = 91) appear on the score of β preferential cleavage:

**Figure:**
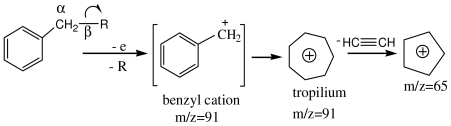


The fragment ion *m*/*z* = 65 is characterized in the spectra of amphetamines by a very low abundance. However, the importance of this fragment results from the fact that it is present in all the spectra of the amphetamines. Thus, we can conclude that the fragment ion *m*/*z* = 65 participates to both the modeling of amphetamines and to the discrimination between amphetamines and nonamphetamines.

Another important fragment is the benzyl cation (*m*/*z* = 91), which is very characteristic to the GC-MS spectra of stimulants. It contributes significantly to the modeling power of the system in the case of the M class and, at same the time, it helps 18PC_IR-MS-ANN to discriminate this class from the classes T and N.

The β cleavage of the aromatic hydrocarbons with an aliphatic side chain result in the fragment ions *m*/*z* = 58 and *m*/*z* = 72, representing conjugated alchilamine fragments (R^+^). The fragment ion *m*/*z* = 58 appears in the GC-MS spectra [23] of the following positives from the training set: methamphetamine, β-phenyletilamine, 1-(3,4-methylenedioxyphenyl)-2-butanamine, 3,4-methylenedioxymethamphetamine, *N*-ethylamphetamine, *N*-methyl-α-phenylethylamine and *N*-n-propylamphetamine, and the fragment ion *m*/*z* = 72 is base ion for the positives: *N*-ethylamphetamine and 3,4-methylenedioxy-*N*-ethylamphetamine. We may conclude that these fragments participate to the modeling of M and T classes, and to the discrimination of these classes from the nonamphetamines class.

The phenyl cation *m*/*z* = 77 has the biggest contribution to PC3, its loading being of 0.21 (Figure 5). The aromatic hydrocarbons with an aliphatic side chain give type α cleavages, and the phenyl cation is obtained:

**Figure:**
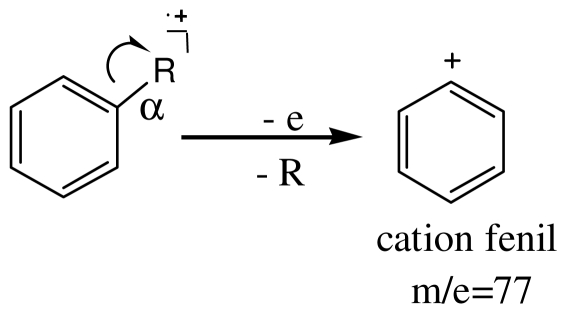


It is worth emphasizing that this fragment characterizes the M and T classes, hereby having a significant contribution to the modeling of these classes.

## 4. Conclusions

The aim of this paper was to adapt chemometrical techniques to differentiate between amphetamines according to their biological activity and identifying the best operating expert system. Building expert systems able to distinguish between amphetamines and nonamphetamines presents the challenge of obtaining a very good modeling of the nonamphetamines, without diminishing the modeling power of the classes of amphetamines (stimulant and hallucinogens). In other words, the challenge is to obtain a very good capacity of the system to classify the majority, if not all the compounds, and at the same time provide the results (the class assignment) with a good speed of work. These challenges were successfully overreached with expert system found to have the best results (18PC_IR-MS-ANN) and which was obtained by optimizing the number of input variables.

As amphetamines are small molecules, small changes in their molecular structure yield significant changes in their GC-FTIR and GC-MS spectra. PCA has been used in order to eliminate the input variables that have a lower contribution to the modeling/discrimination power of the modeled classes. In other words, the redundant information has been eliminated, and only the relevant information was kept for the processing system. In our case, the 18 PCs represent a total of 77% explained variance. The 18PC_IR-MS-ANN network has the best rate of classification and rate of true positives (C = 100% and TP = 100%) and very good result for the rate of true negatives (TN = 92.77%). Finally, we should stress that the input concatenated database that was preprocessed by PCA allows a very efficient automatic determination of the class identity of an unknown compound representing either an amphetamine analogue or a substance with a similar molecular structure. The class identity is attributed to the new compound according to its biological activity, even if the unknown substance is not present in the training database.

## Figures and Tables

**Table 1:** Validation results and explained variance for the 100imp_IR-MS-ANN and the PC-ANN networks.

	**100imp_IR-MS-ANN**	15PC_IR-MS-ANN	16PC_IR-MS-ANN	17PC_IR-MS-ANN	**18PC_IR-MS-ANN**	19PC_IR-MS-ANN	20PC_IR-MS-ANN	21PC_IR-MS-ANN	22PC_IR-MS-ANN
Explained variance (%)		72	73	75	**77**	78	79	81	82
TP (%)	**100**	100	95	100	**100**	100	100	100	100
TN (%)	**93.83**	90.79	89.87	87.5	**92.77**	92.21	93.67	92.31	88.46
FP (%)	**6.17**	9.21	10.13	12.5	**7.3**	7.79	6.33	7.69	11.54
FN (%)	**0**	0	5	0	**0**	0	0	0	0
CC (%)	**95.05**	92.71	90.91	90	**94.17**	93.81	94.95	93.88	90.82
C (%)	**98.05**	93.2	96.12	97.09	**100**	94.17	96.12	95.15	95.15

**Table 2:** The number of unclassified compounds, of false positives and of false negatives obtained by each network.

	**100imp_IR-MS-ANN**	15PC_IR-MS-ANN	16PC_IR-MS-ANN	17PC_IR-MS-ANN	**18PC_IR-MS-ANN**	19PC_IR-MS-ANN	20PC_IR-MS-ANN	21PC_IR-MS-ANN	22PC_IR-MS-ANN
Unclassified	**2**	7	4	3	**0**	6	4	5	5
False positives	**5**	7	8	10	**6**	6	5	6	9
False negatives	**0**	0	1	0	**0**	0	0	0	0

Total	**7**	14	13	13	**6**	12	9	11	14

**Table 3:** Importance of input variables for the 18PC_IR-MS-ANN expert system.

Input name	Importance
PC3	40.9814
PC2	40.8999
PC4	29.7392
PC5	28.8037
PC1	25.8807
PC7	23.7982
PC12	23.1617
PC18	21.7441
PC10	16.1382
PC15	15.9791
PC8	15.2410
PC6	9.1584
PC14	8.5687
PC13	7.5713
PC17	6.8467
PC11	4.4506
PC16	3.5881
PC9	2.2904
